# Profiles of Human Milk Oligosaccharides and Their Relations to the Milk Microbiota of Breastfeeding Mothers in Dubai

**DOI:** 10.3390/nu12061727

**Published:** 2020-06-09

**Authors:** Carole Ayoub Moubareck, Maryam Lootah, Muna Tahlak, Koen Venema

**Affiliations:** 1 College of Natural and Health Sciences, Zayed University, Dubai, UAE; Carole.AyoubMoubareck@zu.ac.ae (C.A.M.); maryam.lootah@moe.gov.ae (M.L.); 2 Latifa Women and Children Hospital, Dubai, UAE; MATahlak@dha.gov.ae; 3 Centre for Healthy Eating & Food Innovation (HEFI), Maastricht University—Campus Venlo, 5928 RC Venlo, The Netherlands

**Keywords:** human milk oligosaccharides, microbiota, breastfeeding mothers, V3-V4 16S rRNA gene, next generation sequencing

## Abstract

The composition of human breast milk is affected by several factors, including genetics, geographic location and maternal nutrition. This study investigated the human milk oligosaccharides (HMOs) of breastfeeding mothers living in Dubai and their relations with the milk microbiota. A total of 30 breast milk samples were collected from healthy Emirati and UAE-expatriates at Latifa Hospital. HMO profiling was performed using UHPLC-MS. Microbiota profiles were determined by sequencing amplicons of the V3-V4 region of the 16S rRNA gene. HMO concentrations were significantly higher in Emirati, and dropped with the lactation period in both groups of mothers. The Le (a^−^b^+^)-secretor (Le^+^Se^+^) type was the most abundant in Dubai mothers (60%), followed by the Le(a^−^b^−^)-secretor (Le^−^Se^+^) type (23%). *Bifidobacterium* and *Lactobacillus* were considerably lower in Dubai-based mothers, while *Pseudomonas* and *Delftia* (*Hydrogenophaga*) were detected at a higher abundance compared to mothers from other countries. Atopobium was correlated with sialyl-lacto-N-tetraose c, *Leptotrichia* and *Veillonella* were correlated with 6’-sialyl-lactose, and *Porphyromonas* was correlated with lacto-N-hexaose. The study highlights the HMO profiles of breastfeeding mothers in Dubai and reveals few correlations with milk microbial composition. Targeted genomic analyses may help in determining whether these differences are due to genetic variations or to sociocultural and environmental factors.

## 1. Introduction

Human milk is known to provide optimal nutrition for the growth and development of infants. Breastfed infants have been shown to have a reduced risk of allergic diseases [1], sudden infant death syndrome, asthma, type 1 and 2 diabetes, gastroenteritis and respiratory tract infections [2]. Analyzing the composition of human milk is therefore essential to understand the associated physiological advantages. Human milk contains bioactive components that are critical for the infant’s immune system, such as cytokines, immunoglobulins, antibodies, hormones and growth factors. Non-specific compounds, such as human milk oligosaccharides (HMOs), whey proteins including lactoferrin, other proteins (such as lysozyme) and an abundant amount of bacteria are also found in human milk [3,4]. Interestingly, HMOs are the third highest component of human milk, preceded by lactose and lipids [5]. In the infant’s intestine, HMOs function similarly to prebiotics, which are non-digestible substances that promote the growth of beneficial bacteria in the colon, thereby conferring a health benefit [6]. HMOs also enhance the infant’s immunity, as they reduce the risk of bacterial, viral and parasitic infections by attaching to the receptors of epithelial cells, thereby decreasing the subsequent adherence of the microorganisms to the epithelial cells, or functioning as decoy receptors [7,8]. In addition, HMOs reduce the expression of pro-inflammatory cytokines by interacting with immune cells [9].

Structurally, HMOs contain a lactose core bound to one or more glucose, galactose, *N*-acetylglucosamine, fucose or sialic acid residues, with *N*-acetylneuraminic acid being the most predominant form of sialic acid [8,9]. Glycosyltransferases attach *N*-acetylglucosamine, galactose, *N*-acetylneuraminic acid and fucose to lactose, the main acceptor molecule in the mammary gland [10]. Fucose residues may be attached to HMO by an α1,2-linkage, which is catalyzed by a fucosyltransferase genetically encoded by the secretor gene (FUT2) or via α1,3- or α1,4-linkages, which are catalyzed by enzymes encoded by the Lewis gene (FUT3) family [11]. Glycosyltransferases are common to all mothers, while fucosyltransferases are not, and their presence is determined by genetics [10].

Based on the presence and the type of fucosylated HMOs, breast milk can be divided into four categories. This variation is based on the fucosyltransferases (FUT2 and/or FUT3) produced by the mother, which are determined genetically and related to the Lewis and secretor type of the mother [12,13]. About 70% of the population in Europe are Lewis (a^−^b^+^) secretors (Le^+^Se^+^), whose milk contains fucosylated oligosaccharides, with (α1,2) and (α1,4) linkages, next to (α1,3) linkages. The breast milk from this group then contains 2′-Fucosyllactose (2′-FL) as well as Lacto-N-fucopentaose II (LNFP II) next to 3′-Fucosyllactose (3′-FL). This group can also produce Difucosyllactose (DFL). The second group, which comprises about 20% of the European population are Lewis (a^+^b^−^) non secretors (Le^+^Se^−^), who produce milk that contains (α1,4) and (α1,3) fucosylated HMOs, but not (α1,2). The milk from this group contains LNFP II and 3′-FL, but does not contain 2′-FL. Due to the inability to synthesize the (α1,2)-linkage, DFL is also absent. The third group are Lewis (a^−^b^−^) secretors (Le^−^Se^+^), whose milk contains (α1,2) and (α1,3) fucosylated HMOs, such as 2′-FL and 3′-FL. LNFP II is absent due to the inability to synthesize (α1,4) linkages. About 10% of the European population belongs to this group. The last group, which comprise the minority (1%) of the population, are Lewis (a^−^b^−^) non secretors (Le^−^Se^−^). Members of this group are able to produce only (α1,3) fucosylated HMOs, which results in the absence of 2′-FL as well as LNFP II and DFL [12,13].

The type of individual HMOs differs based on the secretor status of the mother and their Lewis blood group [10]. Moreover, oligosaccharide composition and amount vary over the course of lactation [14,15]. As milk production matures and volume increases, HMO concentrations decline. One of the studies that used reversed-phase high performance liquid chromatography showed that the HMO concentration in the first 14 weeks of lactation was 9 g/L, and at 1 year postpartum it had gradually decreased to 4 g/L [15]. Another study used high performance anion exchange chromatography coupled to pulsed amperometric detection (HPAEC-PAD), and showed that the highest amount of oligosaccharides was present at day 4 postpartum (20 g/L), which then decreases by about 20% at day 30 of lactation [16]. The milk of mothers delivering preterm infants has higher HMO concentrations than term milk [17]. Additional factors can alter oligosaccharides’ compositions, such as maternal nutrition [18], geographic location [19,20] and the gestational age; even though the effect of the latter has not been accurately proven [10].

The roles of individual HMOs vary, as each type has specific functions and is metabolized by specific bacteria. Particular interrelations between human milk metabolites and gut microbiota were observed [19].

Human breast milk also contains a microbiota. The existence of the human milk microbiota was discovered only about fifteen years ago [21]. Recent advances in the assessment of early host–microbe interactions suggest that early colonisation may have an impact on later health [21,22], and it seems that the strains in breast milk are particularly suited to colonize the infant gut. Several probiotics have been developed based on breast milk isolates of lactic acid bacteria, particularly *Lactobacillus* species [23,24].

The objective of this study was to investigate the levels and types of HMOs in the milk of breastfeeding mothers living in Dubai, and to observe their relations with their milk microbiota.

## 2. Materials and Methods

### 2.1. Setup of Study and Sample Collection

A total of 16 Emirati and 14 UAE expatriate (Dubai-based UAE-expats) breastfeeding mothers were recruited between March and April 2018 in the lactation clinic of Latifa hospital in Dubai. Details on the babies and mothers including age, lacatation stage, birth mode, number of other children and the origin of the expats are provided in Table 1. Milk collection was performed by manual expression after cleaning the mother’s breast with sterile alcohol pads. A total of 10 mL of milk was collected per mother. Samples were then sent to Zayed University, Dubai for storage at −80 °C. These samples were shipped to the Laboratory of Food Chemistry, Wageningen University, Netherlands for HMO analysis and Maastricht University, Venlo campus, Netherlands to determine microbiota composition. None of the mothers received antibiotics at least 3 days prior to human milk sampling, and none of the mothers took pre- or probiotics in their diet for at least a month prior to sampling. Twelve participants took antibiotics between 3 days and one month prior to milk sampling; 18 participants did not take antibiotics at least one month prior to milk sampling.

Human milk collection was approved by the Dubai Scientific Research Ethics Committee of Dubai Health Authority (DSERC-06/2017_05) and was in accordance with the Helsinki Declaration of 1975, as revised in 1983. Written consent forms were signed by the participants prior to sample collection.

### 2.2. HMO Profiling

#### 2.2.1. Sample Pretreatment

Frozen milk samples were thawed by storage at 4 °C overnight. The thawed samples were mixed and two 0.5 mL aliquots were taken. Each aliquot was diluted with the same volume of water. The diluted samples were centrifuged (20,000× *g*, 4 °C, 20 min) to separate the fat from the serum.

#### 2.2.2. Extraction of Milk Oligosaccharides

The extraction of the milk oligosaccharides was performed by solid phase extraction (SPE) using a graphitised carbon solid phase extraction cartridge (Supelclean ENVI-Carb, Supelco, Darmstadt, Germany, 3 mL). The SPE cartridges were first conditioned by passing 1.5 mL of 80% (*v*/*v*) acetonitrile containing 0.1% (*v*/*v*) trifluoroacetic acid (TFA), followed by washing with 1.5 mL of water. An aliquot of 100 µL milk serum was loaded onto the column, followed by 1.5 mL water for washing. Lactose and 3′-fucosyllactose (3′-FL) were eluted using 3 mL 3% (*v*/*v*) acetonitrile (Fraction A). The rest of the oligosaccharides were eluted in 1.5 mL 40% (*v*/*v*) acetonitrile containing 0.05% (*v*/*v*) TFA (Fraction B). Both fractions were dried under a stream of nitrogen and redissolved in 500 µL water.

#### 2.2.3. Analysis of 3′-Fucosyllactose (3′-FL)

Analysis of 3′-FL was performed on the supernatant of fraction A after ten times dilution, using high performance anion exchange chromatography with pulsed amperometric detection (HPAEC-PAD; Dionex ICS5000, Thermo Scientific, Landsmeer, The Netherlands) equipped with CarboPac PA1 column (2 × 250 mm) and a guard column (2 × 50 mm) (Thermo Scientific). The flow rate was kept at 0.3 mL/min. The 3′-FL was eluted in a 4 min isocratic elution of 20 mM sodium acetate in 0.1 M sodium hydroxide. After every analysis, the column was cleaned using 1 M sodium acetate in 0.1 M sodium hydroxide for 5 min, followed by 11 min re-equilibration under the starting condition. Quantification of 3′-FL was performed based on a standard range of 3′-FL from 1 to 20 µg/mL.

#### 2.2.4. Analysis of 2′-FL, DFL, 3′-Sialyllactose (3′-SL), 6′-Sialyllactose (6′-SL), Lacto-N-tetraose (LNT), Lacto-N-neotetraose (LNnT), Lacto-N-fucopentaose I (LNFP I), LNFP II, Lacto-N-fucopentaose V (LNFP V), Lacto-N-difucohexaose (LNDFH), Sialyl-lacto-N-tetraose a (LSTa), Sialyl-lacto-N-tetraose b (LSTb), Sialyl-lacto-N-tetraose c (LSTc), Lacto-N-hexaose (LNH), Fucosyllacto-N-Hexaose-III (FLNH-III) and Lacto-N-neohexaose (LNnH)

Reduction step: the oligosaccharides in Fraction B were reduced by mixing 200 µL Fraction B with 200 µL 0.5 M sodium borohydride, followed by overnight incubation at room temperature. The reduced oligosaccharides were then extracted from the mixture using a graphitised carbon solid phase extraction cartridge (Supelclean ENVI-Carb, Supelco, 3 mL). The SPE cartridges were conditioned and washed as described above. After loading the reduced sample, excess reagent was eluted using 6 mL of water. The reduced oligosaccharides were then eluted in 40% (*v*/*v*) acetonitrile containing 0.05% (*v*/*v*) TFA, dried and redissolved in 400 µL water. Mixtures of oligosaccharide standards (200 µL) containing 20 µg/mL of each standard compound were treated in the same way as the samples.

UHPLC-MS analysis: the samples and standards after reduction were diluted two times, followed by analysis of the oligosaccharides on a Vanquish UHPLC system (Thermo Scientific) equipped with a Hypercarb column (100 × 2.1 mm, Thermo Scientific) maintained at 25 °C. The eluents were UHPLC grade water (Biosolve, Dieuze, France) containing 1% (*v*/*v*) acetonitrile and 0.1% (*v*/*v*) formic acid (Eluent A), and UHPLC grade acetonitrile containing 0.1% (*v*/*v*) formic acid (Eluent B). The elution was started by an isocratic elution of 3% (*v*/*v*) B for 5 min, followed by a 1%/min gradient to 20% (*v*/*v*) B and sequentially 2%/min gradient to 40% (*v*/*v*) B. The flow rate was 0.2 mL/min. Afterwards, the column was cleaned in 100% B for 10 min at 0.3 mL/min, followed by re-equilibration at the starting condition for 10 min at 0.3 mL/min and 11 min at 0.2 mL/min.

Detection of the oligosaccharides was performed using an LTQ VelosPro Mass Spectrometer (Thermo Scientific) set to negative mode, with an m/z range of 300–2000. Capillary temperature was set at 250 °C, and the source heater temperature was 50 °C. The MS was tuned using maltotetraose. Quantification was performed based on the average peak area from standards injected in the beginning, middle and end of the sample analysis.

### 2.3. Breast milk DNA Extraction and Microbial 16S rRNA Gene Sequencing

Genomic DNA extraction was performed using the Quick-DNA™ Fecal/Soil Microbe Miniprep Kit (Zymo Research, Leiden, The Netherlands) according to the manufacturer’s instructions. 16S rRNA gene amplicon libraries for Illumina 2 × 300 bp paired end MiSeq sequencing were generated and sequenced at BaseClear (Leiden, The Netherlands). Briefly, barcoded amplicons from the V3–V4 region of 16S rRNA genes were generated using a 2-step PCR. For this, 10–25 ng genomic DNA was used as the template for the first PCR using the 341F (5′-CCTACGGGNGGCWGCAG−3′) and the 785R (5′-GACTACHVGGGTATCTAATCC−3′) primers appended with Illumina adaptor sequences in a total volume of 50 μL. PCR products were purified (QIAquick PCR Purification Kit, Qiagen, Venlo, The Netherlands). Subsequently, the size of the PCR products was checked on a fragment analyzer (Advanced Analytical, Ankeny, IA, USA) and quantified by fluorometric analyses. These purified PCR products were used for the second PCR in combination with sample-specific unique barcoded primers (Nextera XT index kit, Illumina, Eindhoven, The Netherlands). Subsequently, PCR products were purified, checked on a Fragment analyzer and quantified as described above. Then samples were multiplexed, clustered and sequenced on an Illumina MiSeq. The raw data were analyzed with the Illumina CASAVA pipeline (v1.8.3; Illumina) with demultiplexing based on the unique sample-specific barcodes.

Sequences were converted into FASTQ files using BCL2FASTQ pipeline version 1.8.3. The quality cut was applied based on the quality level of the Phred (Phred quality score). The Quantitative Insights Into Microbial Ecology (QIIME) software package (1.9.0) was used for microbial analyses [25]. The sequences were classified using Greengenes (version 13.8) as a reference 16S rRNA gene database. Linear discriminant analysis effect size (LEfSe) [26] was used to find biomarkers between groups using relative abundances from the operational taxonomic unit (OTU) tables generated in QIIME.

### 2.4. Statistical Analysis

The software package R (3.5.0) (R Core Team, http://www.R-project.org/) was used to determine correlations between OTUs and variables in the study. These statistical analyses were performed with RStudio. A Spearman correlation was calculated between the relative abundance of OTUs and the continuous variables (e.g., HMO concentrations). A Kruskal–Wallis correlation was determined between the relative abundance of OTUs and non-continuous values (e.g., Emirati vs. UAE-expats). Analyses were corrected for multiple comparison by using the false discovery rate (FDR), and *q*-values (adjusted *p*-values) were considered significantly different at a strict value < 0.05.

## 3. Results

### 3.1. HMO Profiling

A total of 17 HMOs were identified in the human milk of breastfeeding mothers in the UAE. They are reported for each individual in Table S1. The total amount of HMOs detected in the 30 samples, as presented in Table 2, was between 2.3 and 9.4 mg/mL, with a total of 4.9–9.4 mg/mL for <1 month of lactation (*n* = 21) and 2.3–6.5 mg/mL for >1 month of lactation (*n* = 9).

Table 2 shows significant differences as a function of the origin of the breastfeeding mothers (Emirati or UAE-expats), and lactation period (<1 month vs. >1 month), and two of the Lewis/secretor types (Le^+^Se^−^ and Le^−^Se^−^). The HMO 6′-SL was higher in Emirati (0.292 mg/mL ± 0.242 for UAE-expats vs. 0.491 mg/mL ± 0.196 for Emirati; *p*-value 0.043; Figure S1A). Likewise, LSTc was higher in Emirati (0.256 mg/mL ± 0.267 for UAE-expats vs. 0.530 mg/mL ± 0.339 for Emirati; *p*-value 0.024; Figure S1B). Moreover, the total HMOs (sum of the 17 HMOs determined) were significantly higher in Emirati (5.568 mg/mL ± 2.065 for UAE-expats vs. 7.279 mg/mL ± 1.410 for Emirati; *p*-value 0.019; Figure S1C).

The average concentration of total HMOs dropped from 7.333 mg/mL ± 1.210 at <1 month of lactation (*n* = 21) to 4.491 mg/mL ± 1.878 at >1 month of lactation (*n* = 9; *p*-value 0.002). Except for 3′-FL and DFL (which increased, but not significantly), and 3′-SL and LSTb (which remained at the same concentration), all other HMOs dropped in concentration, of which the following HMOs decreased significantly: 6′-SL (0.515 mg/mL ± 0.142 for <1 month of lactation vs. 0.088 mg/mL ± 0.107 for > 1 month of lactation; *p*-value 4.3 × 10^−8^), LNT/LNnT (1.673 mg/mL ± 0.756 vs. 0.790 mg/mL ± 0.501; *p*-value 0.002), LNFP-II (0.390 mg/mL ± 0.472 vs. 0.135 mg/mL ± 0.159; *p*-value 0.042), LNFP-V (0.072 mg/mL ± 0.082 vs. 0.027 mg/mL ± 0.022; *p*-value 0.032), LSTc (0.537 mg/mL ± 0.293 vs. 0.089 mg/mL ± 0.190; *p*-value 8.8 × 10^−5^), LNH (0.202 mg/mL ± 0.219 vs. 0.049 mg/mL ± 0.067; *p*-value 0.009), FNLH-III (0.378 mg/mL ± 0.248 vs. 0.097 mg/mL ± 0.080; *p*-value 1.1 × 10^−4^) and LNnH (0.061 mg/mL ± 0.059 vs. 0.026 mg/mL ± 0.023; *p*-value 0.032).

The grouping of the sampled population based on Lewis-secretor type is shown in Table 3. The grouping was performed based on the presence of 2′-FL and LNFP-II. A total of 18 participants (60% of the population) were Le (a^−^b^+^)-secretor (Le^+^Se^+^). The second most abundant type in Dubai mothers was Le(a^−^b^−^)-secretor (Le^−^Se^+^) (23%). The non-secretor group was about equally divided between Le(a^+^b^−^) (Le^+^Se^−^) and Le(a^−^b^−^) non-secretors (Le^−^Se^−^), with three and two participants among the total 30 participants, respectively.

### 3.2. Microbiota Analysis

**Figure 1:**
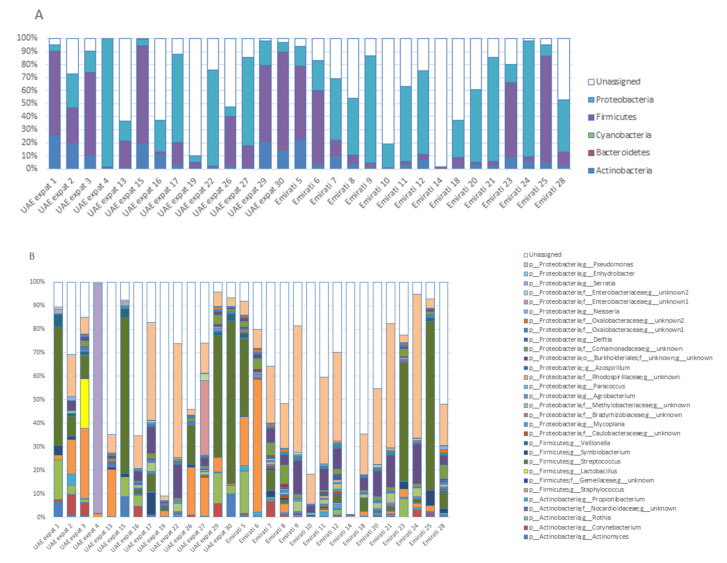
Phyla (**A**) and genera (**B**) present in the breast milk of breastfeeding women in Dubai. Operational taxonomic units (OTUs) are plotted when present at at least 0.5% abundance in one of the individuals. OTUs < 0.5% abundance, or of which the phylogeny could not be determined, are grouped under ‘unassigned’.

A total of 109 genera were detected. Figure 1 shows phylum abundances (Figure 1A) and genera abundances (Figure 1B) for the detected OTUs, with an abundance of at least 0.5% in one of the breastfeeding mothers in Dubai. Other OTUs (<0.5%) and unassigned OTUs are grouped under ‘unassigned’ (see Table S2 for full data set). In one of the Emirati mothers (normal delivery, no antibiotic use), an unknown OTU made up most of the microbiota (98.7%).

The relative abundance (RA) of bifidobacteria was quite low (average 0.08%; range 0–1.5% for all mothers; average 0.02% for Emirati, 0.15% for UAE-expats), whereas lactobacilli were in the top ten most abundant for UAE-expats (average RA 1.54%, due to a 20.8% abundance in one of the mothers; Figure 1B), whereas they were low in Emirati (average RA 0.08%). They were co-abundant when they were present in a sample (Figure S2).

**Figure 2:**
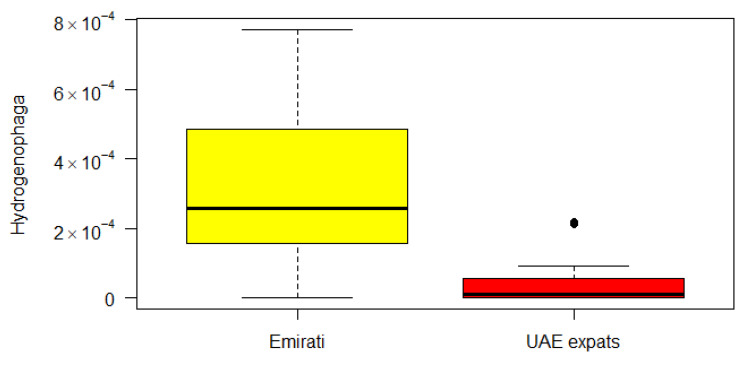
Boxplot of the difference between Emirati and UAE-expats in *Hydrogenophaga*. Yellow: Emirati; red: UAE-expats.

The genus *Hydrogenophaga* (of the beta-Proteobacteria) was significantly different between the Emirati and UAE-expats on the basis of the Kruskal–Wallis correlation (Figure 2).

**Figure 3:**
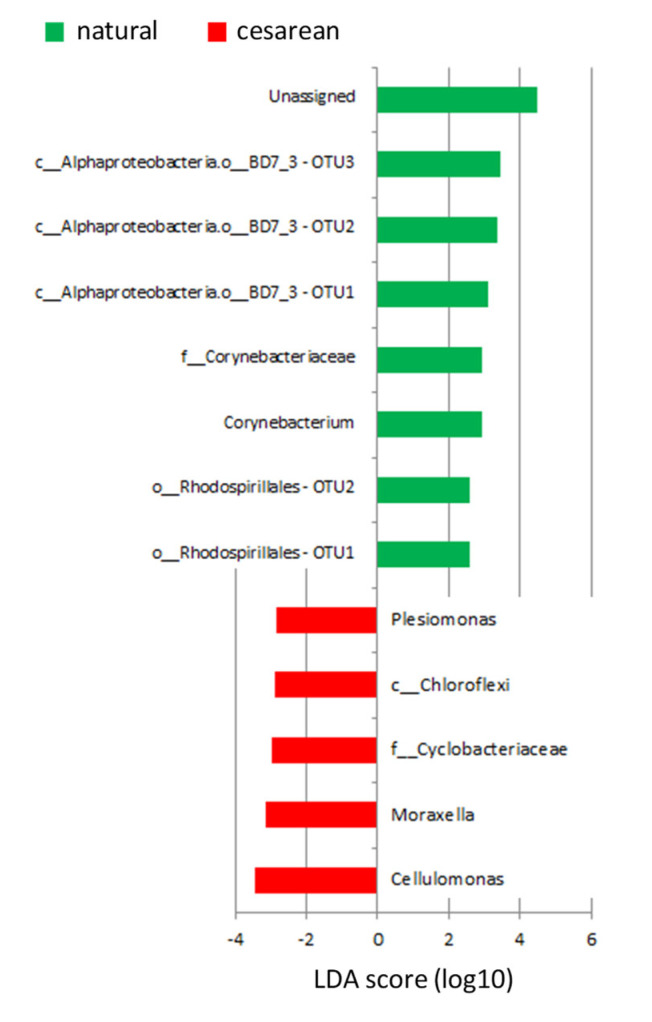
Plot of OTUs that are different between mode of delivery, as identified by LEfSe.

There were no correlations between OTUs and the weight of the mothers, their age, use of antibiotics, length of pregnancy, weight or gender of the offspring, whether the children were born at full term or not, or the total number of children of the mothers. Several correlations were found with LEfSe between mode of delivery and several OTUs (Figure 3). Irrespective of whether the mothers were Emirati or UAE-expats, an unassigned taxa, three unidentified taxa of the BD7_3 order of alpha Proteobacteria, an OTU within the Corynebacteriaceae family, *Corynebacterium* and two unidentified taxa of the Rhodospirillales order were more abundant in mothers that delivered the baby via natural birth. On the other hand, *Cellulomonas*, *Moraxella*, an unidentified taxa of the Cyclobacteriaceae family, an unidentified taxa of the Chloroflexi order and *Plesiomonas* were more abundant in mothers that delivered the baby via C-section.

The Spearman correlation analysis showed correlations between 5 OTUs (*Atopobium*, *Lysobacter*; a genus of the family Marinilabiaceae; *Moryella* and *Oribacterium*) and length of lactation. When plotting those OTUs that were significantly correlated with lactation period, however, these correlations seemed to be mostly driven by one or a few data-points (Figure S3), and the biological relevance needs to be further established. However, the data suggests that those HMOs that are high in concentrations early in lactation (Table 2) may favor these 5 OTUs, which then reduce in abundance later in lactation, when those HMO concentrations decline.

**Figure 4:**
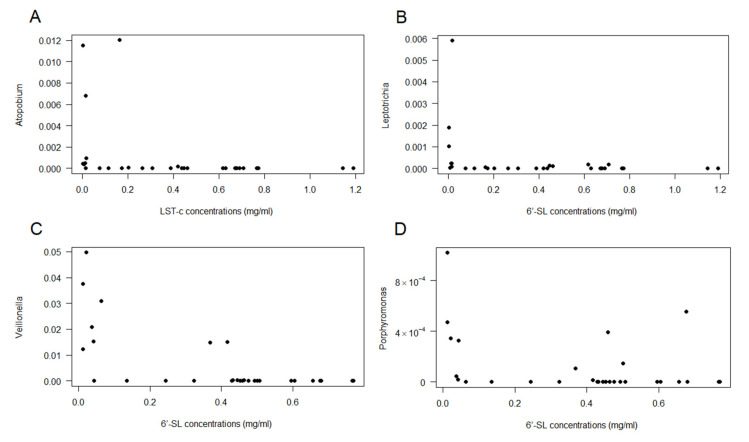
Spearman correlations between OTUs and individual HMO. (**A**): *Atopobium* vs. LSTc; (**B**): *Leptotrichia* vs. 6′-SL; (**C**): *Veillonella* vs. 6′-SL; and (**D**): *Porphyromonas* vs. LNH.

Given the large interindividual variation in breastmilk microbiota composition, there were only a few OTUs that correlated with the presence of individual HMOs (Figure 4). *Atopobium* was negatively correlated with LSTc (Figure 4A), *Leptotrichia* (Figure 4B) and *Veillonella* (Figure 4C) were negatively correlated with 6′-SL, and *Porphyromonas* was negatively correlated with LNH (Figure 4D). None of the OTUs positively correlated with any of the HMOs.

## 4. Discussion

Breastfeeding is the natural way of feeding an infant, providing the ideal balance of nutrients, including HMOs, of which the amount and composition vary substantially between lactating women depending on factors such as genetics, ethnicity and geographic location. In this study, to investigate whether the observed profiling in HMOs was typical for Emirati mothers, HMO profiles of UAE-expat mothers living in Dubai were also determined. Some differences were observed between the two groups of mothers, although the comparison between these groups is weakened by the fact that expats came from different countries (Ethiopia (*n* = 1); India (*n* = 6); Iran (*n* = 1); Omani (*n* = 1); Switzerland (*n* = 1); Syria (*n* = 1); Canada (*n* = 1) and Yemen (*n* = 2)). Comparing the Emirati (*n* = 16) with the expats originating from India (*n* = 6) still gives a significant difference for 6′-SL (*p* = 0.018), but only shows a trend for LSTc and total HMOs (*p* = 0.1 for both). McGuire et al. have previously highlighted that HMO profiles differ in populations, and found that concentrations of 6′-SL and LSTc were higher in milk that was produced by urban Etiopian mothers than by rural Etiopian mothers [20]. Other studies have also shown that the amount of HMO in milk can be different between women and also during different stages of lactation [27,28]. We have observed in this work that concentrations of HMOs decline with length of lactation. The total amount of HMOs detected in the 30 samples decreased from a total of 4.9–9.4 mg/mL for < 1 month of lactation [*n* = 21] to 2.3–6.5 mg/mL for > 1 month of lactation [*n* = 9]. Previous results of HMO concentrations in breast milk until one month after delivery, measured using the same method (UHPLC-MS), showed values between 2.0 and 6.5 mg/mL [29]. Compared to the values analysed using capillary electrophoresis-laser-induced fluorescence in recently published literature [27], these values were in the lower range. In the same literature, however, it was shown that the HMO concentrations decreased with longer lactation periods, although the exact lactation period for these samples is unknown.

Besides this, mothers can synthesize various HMOs depending on their Lewis system and secretory status. Like in the European population, most (60%) of the population living in Dubai were Le (a^−^b^+^)-secretors (Le^+^Se^+^). The second most abundant type in Dubai mothers was Le(a^−^b^−^)-secretor (Le^−^Se^+^) (23%), whereas the second most abundant in the European population was Le (a^+^b^−^) non secretor (Le^+^Se^−^) [13]. The non-secretor group was about equally divided between Le(a^+^b^−^) (Le^+^Se^−^) and Le(a^−^b^−^) non-secretors (Le^−^Se^−^), with three and two participants among the total 30 participants, respectively. There was not a lot of difference for the other types, although Le(a^−^b^+^) secretors are more prevalent in the non-Emirati (10 of 14 vs. 8 of 16 in the Emirati). This composition of the population, however, may not be representative of the whole Middle East population because of the small number of samples analysed.

An attempt was made to correlate the concentrations of HMOs to the milk microbiota. *Atopobium, Leptotrichia*, *Veillonella* and *Porphyromonas* negatively correlated with specific HMOs, and it could be hypothesized that, because they were able to consume that particular HMO, they proliferated in the mammary gland, but this remains to be investigated.

Besides this, correlations were found between mode of delivery and several OTUs. *Corynebacterium* was more abundant in mothers that had a natural delivery, while *Cellulomonas*, *Moraxella* and *Plesiomonas* were more abundant in mothers that had a C-section delivery. None of the species identified to the genus level have been correlated with mode of delivery before. The genus *Corynebacterium* contains both pathogenic and non-pathogenic species [30], and has been isolated from breast milk before [31]. *Cellulomonas* belongs to the phylum Actinobacteria, and includes the only known and reported cellulolytic facultative anaerobes [32]. Why that would be relevant in breastmilk is unclear. It has been found in breastmilk before, but was considered a contamination in that study [33]. *Moraxella* has also been found in the breastmilk of healthy mothers before [31]. Lastly, *Plesiomonas,* particularly the *shigelloides* species, is usually correlated with gastroenteritis. As far as we can tell it has never been found in breastmilk before. Antibodies against *P. shigelloides* have been found in mothers that have been infected with this species [34], and this is thought to protect the infant against infection by *P. shigelloides.* We did not test for antibody titers.

The genus *Hydrogenophaga* (of the beta-Proteobacteria) was significantly different between the Emirati and UAE-expats on the basis of the Kruskal–Wallis correlation (Figure 2). The genus *Hydrogenophaga* belongs to the family Comamonadaceae, a large and diverse bacterial family belonging to the order Burkholderiales, and currently comprises over 100 species in 29 genera. *Hydrogenophaga* has been found to be in greater abundances in breast cancer tissue compared with the corresponding normal tissue, along with a variety of other bacteria [35], but has been found in normal tissue as well [35].

Classical genera used as probiotics are *Bifidobacterium* and *Lactobacillus*. Relative abundances of these genera were considerably lower in Dubai-based mothers than in mothers from other continents (Africa, the Americas and Europe), which ranged from 3.7–8.3% for *Lactobacillus* and 0.8–2.5% for *Bifidobacterium* [19].

**Figure 5:**
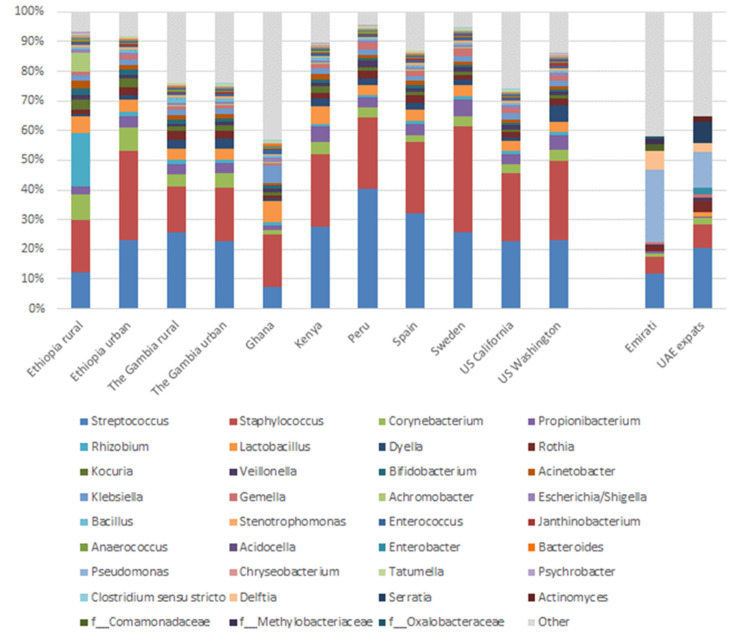
Mean relative abundances of an aggregation of the ten most abundant genera in milk in each cohort.

A recent paper by Lackey et al. [19] compared the milk microbiota of eight different countries globally (and within two countries the differences between rural and urban, and within the US between California and Washington). Based on the top ten most abundant OTUs, the Emirati and UAE-expats data has been compared to the data of Lackey et al. and is shown in Figure 5. Some differences can be observed between the mothers living in Dubai and the other countries. Most notable is a much higher abundance of *Pseudomonas* (24.2% in Emirati, 11.8% in UAE-expats vs. 0.2–0.4% in the other countries) and *Delftia* (not within the top ten in the other countries; average 6.4% in Emirati, 3.2% in UAE-expats). Proteobacteria, which includes *Pseudomonas* and *Delftia* (and *Hydrogenophaga,* which was significantly different between the two groups, as discussed above), is the most abundant phylum represented in breast tissue [36]. Proteobacteria are also the principal phylum in human milk [37], with many of the same bacteria that were detected in tissue also being present in milk, suggesting that the tissue microbiota could be a source of these bacteria in human milk.

This initial study of the composition of HMOs and milk microbiota of UAE-based mothers has several limitations. The number of included volunteers was low. UAE-expats came from several different origins, which may have influenced the variation within that group. Both mothers giving normal birth and giving birth by cesarean section were included. With cesarian section, prophylactic antibiotics were used. However, the first human milk sampling in this subgroup was after 6 days. The range of lactation periods was relatively large, although this allowed us to observe correlations between lactation period and the presence of several OTUs (Figure S3).

## 5. Conclusions

The objectives of this study were to determine the predominant types of HMOs and microbiota profiles in the milk of lactating mothers in Dubai, as there are no data available regarding this topic locally or regionally. The results show high variability in HMOs and OTUs in the Dubai-based mothers. The only taxon that was significantly different between the two groups (Emirati vs. UAE-expats) was *Hydrogenophaga*. In comparison to other mothers from other continents (Africa, the Americas, Europe), Dubai mothers contained on average more proteobacteria. Targeted genomic analyses may help to determine whether these differences are due to genetic variations or to sociocultural and environmental factors.

## Figures and Tables

**Table 1:** Characteristics of the Emirati (*n* = 16) and UAE expat (*n* = 14) breastfeeding mothers and their babies.

	Mother						Baby			
Sample #	Origin	Weight	Age	Type of Delivery	Lactation Period	Length of Pregnancy	Weight (Baby)	Gender	Full Term or Premature	Number of Children
Emirati 5	Emirati	84 kg	40	Cesarean	5 months	38 W	2.8 kg	F	Full Term	2nd
Emirati 6	Emirati	80 kg	29	Normal	7 days	32 W	1.6 kg	F	Moderate to Late Premature	3rd
Emirati 7	Emirati	78 kg	34	Normal	3 days	38 W	3.0 kg	M	Full Term	3rd
Emirati 8	Emirati	59 kg	26	Normal	3 days	38 W	2.7 kg	M	Full Term	2nd
Emirati 9	Emirati	72 kg	36	Cesarean	11 days	31 W	1.9 kg	M	Very Premature	7th
Emirati 10	Emirati	156 kg	38	Normal	4 days	38 W	3.2 kg	F	Full Term	2nd
Emirati 11	Emirati	70 kg	21	Normal	5 days	36 W	2.4 kg	M	Full Term	1st
Emirati 12	Emirati	100 kg	24	Normal	4 days	38 W	3.5 kg	M	Full Term	2nd
Emirati 14	Emirati	53 kg	25	Normal	10 days	36 W + 6 D	2.8 kg	M	Moderate to Late Premature	1st
Emirati 18	Emirati	77 kg	24	Normal	5 days	38 W	2.8 kg	F	Full Term	1st
Emirati 20	Emirati	49–50 kg	31	Normal	14 days	38 W	2.3 kg	F	Full Term	4th
Emirati 21	Emirati	90 kg	23	Cesarean	7 days	38 W	3.1 kg	F	Full Term	1st
Emirati 23	Emirati	53 kg	26	Cesarean	14 days	36 W + 6 D	2.4 kg	M	Moderate to Late Premature	1st
Emirati 24	Emirati	97 kg	38	Normal	4 months	36 W	3.3 kg	M	Moderate to Late Premature	4th
Emirati 25	Emirati	80.5 kg	37	Normal	1 month	40 W	3.6 kg	M	Full Term	5th
Emirati 28	Emirati	78 kg	37	Normal	10 days	38 W	3.1 kg	F	Full Term	1st
UAE expat 1	Syria	66 kg	22	Normal	1 yr + 3 months	38 W	3.7 kg	F	Full Term	1st
UAE expat 2	Switzerland	51.5 kg	39	Normal (assisted suction cup)	8.5 months	40 W	3.2 kg	M	Full Term	3rd
UAE expat 3	UK/Canada	59 kg	38	Normal	7 months	39 W + 2 D	3.5 kg	M	Full term	3rd
UAE expat 4	Indian	68.5 kg	33	Cesarean	48 days	28 W (Twins)	1.2 kg/710 g	M/M	Very Premature	1st
UAE expat 13	Indian	51 kg	29	Cesarean	6 days	33 W	1.8 kg	F	Moderate to Late Premature	1st
UAE expat 15	Indian	59 kg	32	Cesarean	2 yr	40 W + 2 D	3 kg	F	Full Term	1st
UAE expat 16	Iran	70 kg	27	Normal	11 days	38 W	3.3 kg	M	Full Term	1st
UAE expat 17	Ethiopia	71.5 kg	38	Cesarean	8 days	35 W	2.5 kg	M	Moderate to Late Premature	7th
UAE expat 19	Omani	60.5 kg	33	Normal	13 days	39 W	2.2 kg	F	Full Term	3rd
UAE expat 22	Indian	82 kg	29	Normal	7 days	39 W+ 3 D	3.2 kg	F	Full Term	2nd
UAE expat 26	Yemeni	58 kg	20	Normal	10 days	37 W	2.5 kg	F	Full Term	1st
UAE expat 27	Indian	71 kg	27	Cesarean	78 days	27 W + 4 D	750 g	F	Extremely Premature	1st
UAE expat 29	Yemeni	44 kg	24	Normal	13 days	39 W	3.0 kg	M	Full Term	3rd
UAE expat 30	Indian	74 kg	31	Cesarean	4 months	41 W + 6 D	3.7 kg	M	Full Term	1st

UAE = United Arab Emirates; W = weeks; D= days; M = male; F = female.

**Table 2:** Significant differences in HMO concentrations (mg/mL) as a function of the nationality of the breastfeeding mothers (Emirati or UAE-expats), lactation period (< 1 month vs. > 1 month), and two of the Lewis/secretor types (Le^+^Se^−^ and Le^−^Se^−^).

				Lactation		Lewis a^+^b^−^ Non-Secretor		Lewis a^−^b^−^ Non-Secretor	
		Emirati	UAE Expats	<1 Month	>1 Month	No	Yes	No	Yes
6′-SL	average	0.471	0.292	0.515	0.088				
	SD	0.196	0.242	0.142	0.107				
	*p*-value	0.043		4.32 × 10^−8^					
LSTc	average	0.530	0.256	0.537	0.089				
	SD	0.339	0.267	0.293	0.190				
	*p*-value	0.024		8.83 × 10^−5^					
total HMO	average	7.279	5.568	7.333	4.491				
	SD	1.410	2.065	1.120	1.878				
	*p*-value	0.019		0.002					
LNFP-II	average			0.390	0.135			0.335	0.427
	SD			0.472	0.159			0.009	0.009
	*p*-value			0.042				4.91 × 10^−4^	
LNFP-V	average			0.072	0.027	0.063	0.075		
	SD			0.082	0.022	0.019	0.016		
	*p*-value			0.032		0.035			
LNnH	average			0.061	0.026			0.054	0.054
	SD			0.059	0.023			0.008	0.002
	*p*-value			8.83 × 10^−5^				1.67 × 10^−4^	
LNT/LNnT	average			1.673	0.790				
	SD			0.756	0.501				
	*p*-value			0.002					
LNH	average			0.202	0.219				
	SD			0.049	0.067				
	*p*-value			0.009					
FNLH-III	average			0.378	0.248				
	SD			0.097	0.080				
	*p*-value			1.07 × 10^−4^					
LSTb	average					0.105	0.058		
	SD					0.040	0.008		
	*p*-value					3.36 × 10^−5^			
DFL	average							0.087	0.078
	SD							0.015	0.015
	*p*-value							0.028	
LNDFH	average							0.519	0.434
	SD							0.042	0.015
	*p*-value							4.98 × 10^−6^	

**Table 3:** Grouping of the sampled breastfeeding mothers based on Lewis-secretor type.

Country of Origin		2′-FL	LNFP II	Le(a^−^b^+^) Secretor	Le(a^+^b^−^) Non-Secretor	Le(a^−^b^−^) Secretor	Le(a^−^b^−^) Non-Secretor
UAE	Emirati	+	+	x			
UAE	Emirati	+	+	x			
UAE	Emirati	+	+	x			
UAE	Emirati	+	+	x			
UAE	Emirati	+	+	x			
UAE	Emirati	+	+	x			
UAE	Emirati	+	+	x			
UAE	Emirati	+	+	x			
UAE	Emirati	+	+	x			
UAE	Emirati	+	+	x			
UAE	Emirati	+	+	x			
Syria	UAE expat	+	+	x			
Switzerland	UAE expat	+	+	x			
Indian	UAE expat	+	+	x			
Indian	UAE expat	+	+	x			
Iran	UAE expat	+	+	x			
Yemeni	UAE expat	+	+	x			
Yemeni	UAE expat	+	+	x			
UAE	Emirati	+	-			x	
UAE	Emirati	+	-			x	
Canada	UAE expat	+	-			x	
Indian	UAE expat	+	-			x	
Ethiopia	UAE expat	+	-			x	
Indian	UAE expat	+	-			x	
Indian	UAE expat	+	-			x	
UAE	Emirati	-	+		x		
UAE	Emirati	-	+		x		
Omani	UAE expat	-	+		x		
UAE	Emirati	-	-				x
Indian	UAE expat	-	-				x
	number			18	3	7	2
	percentage of total			60	10	23	7

## Supplementary Materials

The following are available online at https://www.mdpi.com/2072-6643/12/6/1727/s1, Figure S1: Boxplots of the significantly different HMOs between Emirati and UAE-expats. (A) 6′-SL; (B) LSTc; and (C) total HMO, Figure S2: Correlation and anti-correlation between the presence of the operational taxonomic units (OTUs) in breastmilk at the genus level, Figure S3. Spearman correlations between 5 OTUs and lactation period. A: *Atopobium*, B: *Lysobacter*; C: a genus of the family Marinilabiaceae; D: *Moryella*, and E: *Oribacterium*, Table S1: Individual data of the Emirati (*n* = 16) and UAE expat (*n* = 14) breastfeeding mothers regarding HMOs concentrations (mg/mL) present in breastmilk, Table S2: Relative abundance of all observed OTUs for the individual mothers.
