# Supplementary material for: Profiles of Human Milk Oligosaccharides and Their Relations to the Milk Microbiota of Breastfeeding Mothers in Dubai
Source: Nutrients. 2020 Jun 9;12(6):1727. doi: 10.3390/nu12061727 (PMC7353065; doi:10.3390/nu12061727)
Supplement: Supplementary file 1 [file nutrients-12-01727-s001.zip › Supplemental Figures - final.docx]

**Supplementary Materials:**


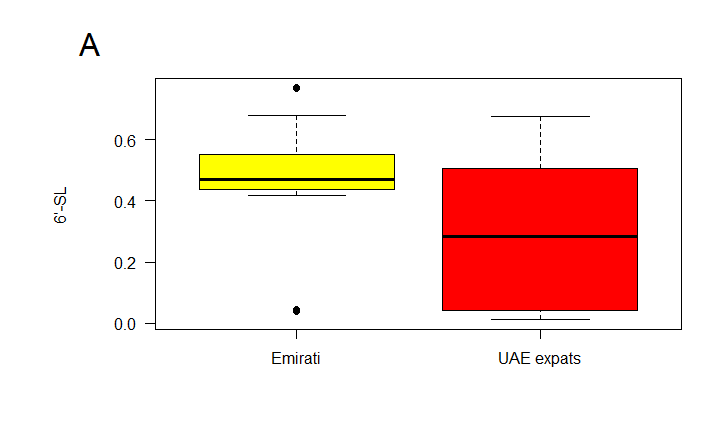


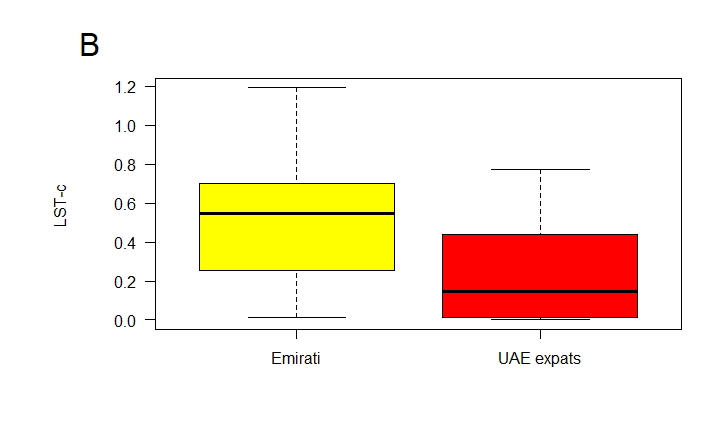


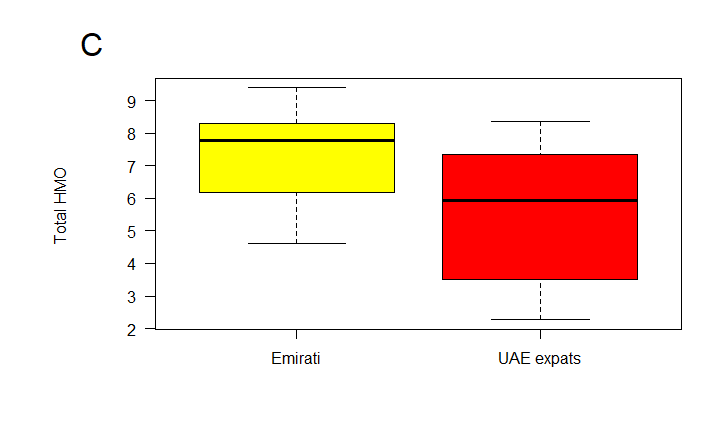


**Figure S1.** Boxplots of the significantly different HMOs between Emirati and UAE-expats. (**A**) 6′-SL; (**B**) LSTc; and (**C**) total HMO. Yellow: Emirati; red: UAE expats.


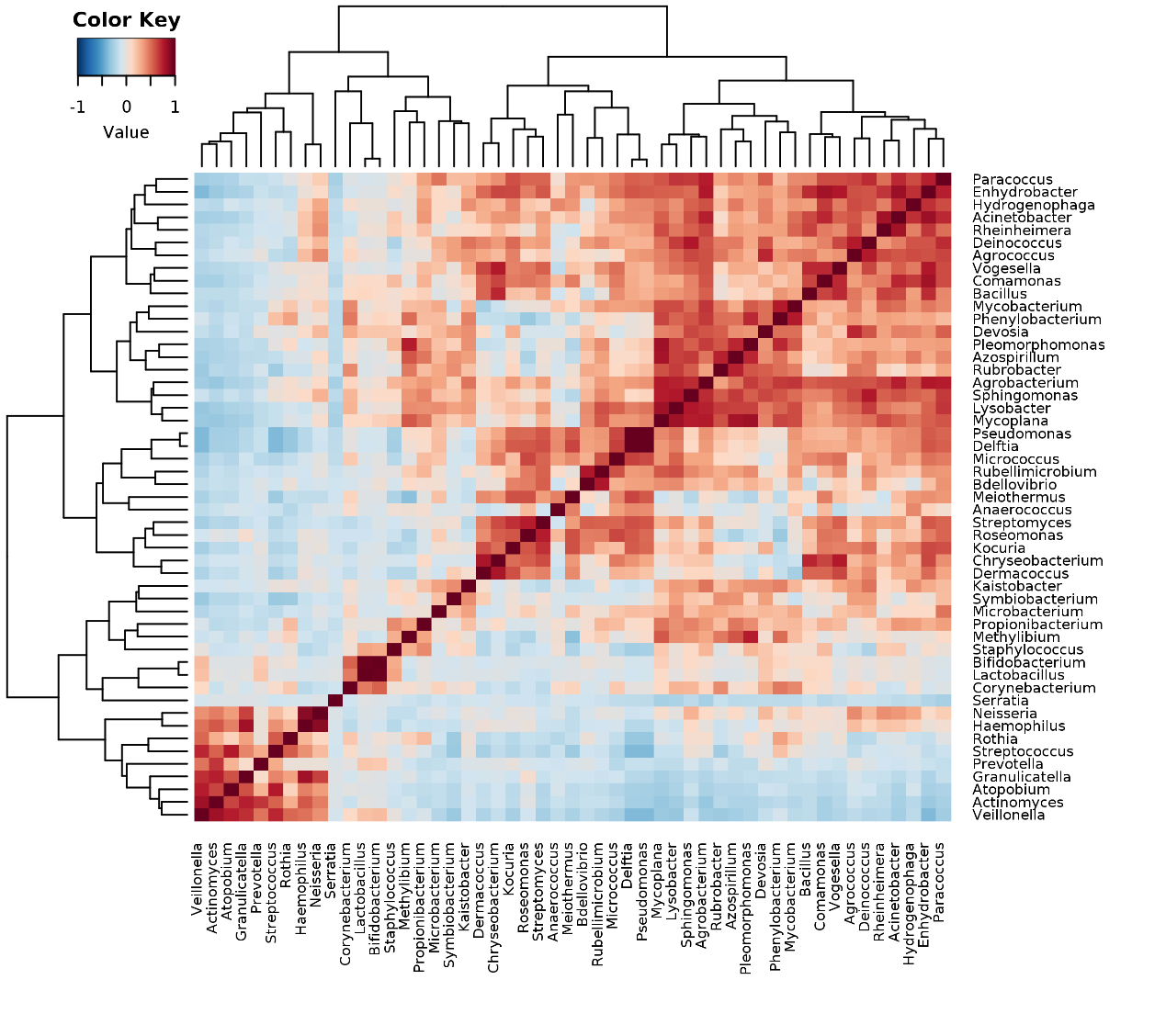


**Figure S2.** Correlation and anti-correlation between the presence of the operational taxonomic units (OTUs) in breastmilk at the genus level.


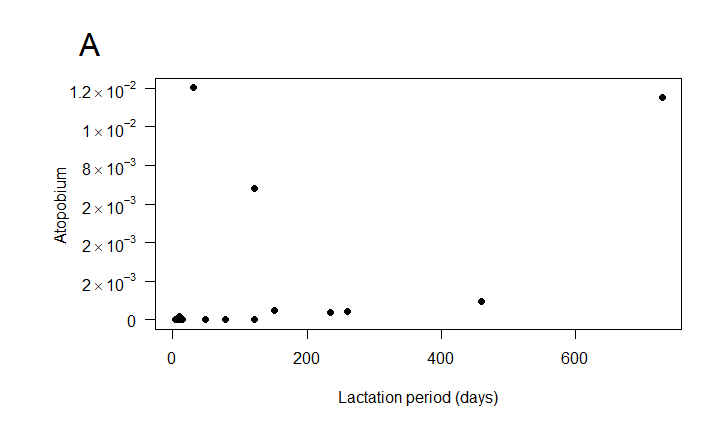


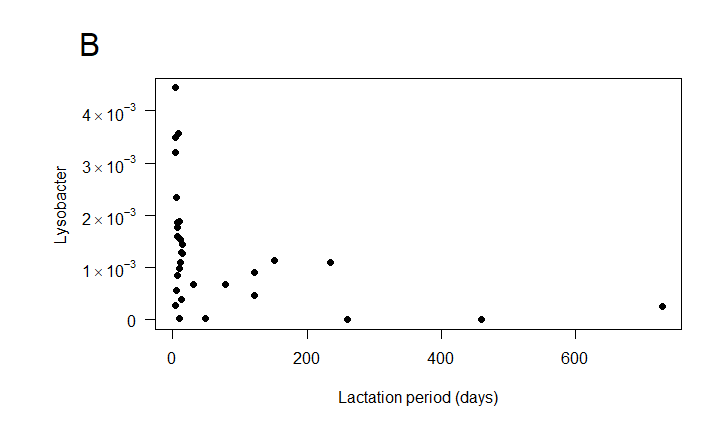


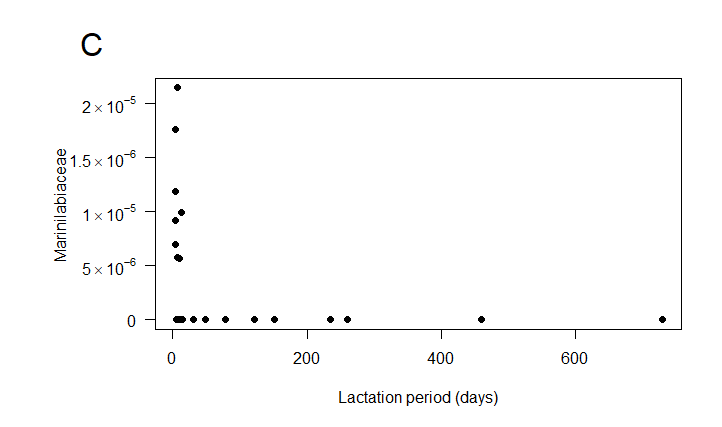


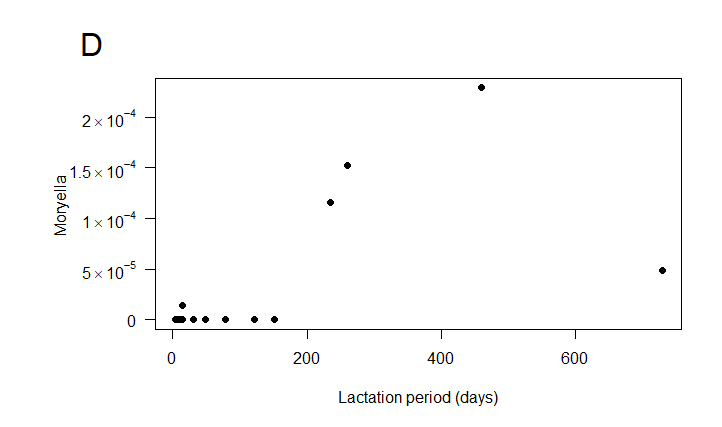


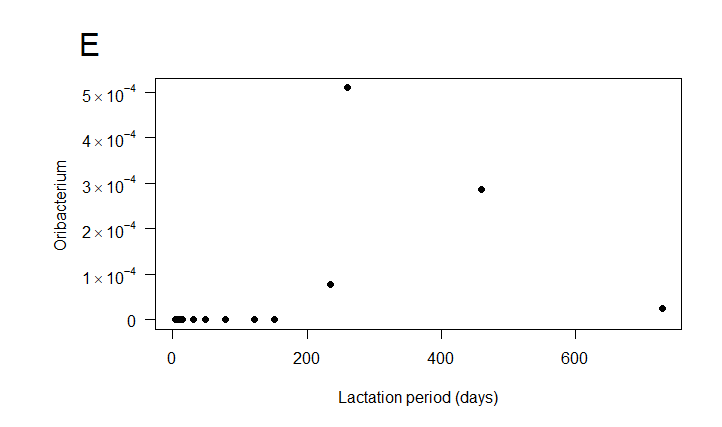


**Figure S3.** Spearman correlations between 5 OTUs and lactation period. A: *Atopobium*, B: *Lysobacter*; C: a genus of the family Marinilabiaceae; D: *Moryella*, and E: *Oribacterium*.
